# OCLI-023, a Novel Pyrimidine Compound, Suppresses Osteoclastogenesis *In Vitro* and Alveolar Bone Resorption *In Vivo*

**DOI:** 10.1371/journal.pone.0170159

**Published:** 2017-01-13

**Authors:** Hye Jung Ihn, Taeho Lee, Ju Ang Kim, Doohyun Lee, Nam Doo Kim, Hong-In Shin, Yong Chul Bae, Eui Kyun Park

**Affiliations:** 1 Department of Oral Pathology and Regenerative Medicine, School of Dentistry, IHBR, Kyungpook National University, Daegu, Republic of Korea; 2 College of Pharmacy, Research Institute of Pharmaceutical Sciences, Kyungpook National University, Daegu, Republic of Korea; 3 New Drug Development Center, Daegu-Gyeongbuk Medical Innovation Foundation, Daegu, Republic of Korea; 4 Department of Oral Anatomy and Neuroscience, School of Dentistry, Kyungpook National University, Daegu, Republic of Korea; Universite de Nantes, FRANCE

## Abstract

An abnormal increase in osteoclast differentiation and activation results in various bone-resorptive diseases, including periodontitis, rheumatoid arthritis, and osteoporosis. Chemical compounds containing pyrimidine ring have been shown to regulate a variety of biological processes. Therefore, in order to identify an antiresorptive agent, we synthesized a series of pyrimidine ring-containing chemical compounds, and found that OCLI-023 suppressed the differentiation and activation of osteoclasts *in vitro*. OCLI-023 directly inhibited receptor activator of nuclear factor-κB ligand (RANKL)-induced differentiation of bone marrow macrophages into osteoclasts, without a cytotoxic response. OCLI-023 also downregulated the RANKL-induced mRNA expression of osteoclast markers as well as inhibited the formation of actin rings and resorption pits. OCLI-023 attenuated the RANKL-induced activation of c-Jun N-terminal kinase and nuclear factor kappa-light-chain-enhancer of activated B cell signaling pathways. In a mouse model of periodontitis, ligature induced an increase of distance between cementoenamel junction (CEJ) and alveolar bone crest (ABC) in the second molar, and OCLI-023 significantly reduced it. Histological analysis showed ligature-induced increase of osteoclast numbers was also significantly reduced by OCLI-023. These data demonstrated the inhibitory effect of OCLI-023 on osteoclast differentiation and activity of osteoclasts *in vitro*, as well as on ligature-induced bone loss *in vivo*, and OCLI-023 can be proposed as a novel anti-resorptive compound.

## Introduction

Bones are responsible for supporting teeth and the whole body and repetitively undergo a resorption and formation cycle to maintain the quality and mass throughout the lifetime [1]. Osteoblasts, generated from mesenchymal stem cells, synthesize and mineralize the bone matrix, while osteoclasts are formed by the cellular fusion of monocyte/macrophage-lineage cells and resorb mineralized bone tissue [2, 3]. Accurate regulation of bone formation and bone resorption is a key factor affecting normal bone remodeling. Abnormal bone remodeling, caused by the disproportionately increased bone destruction, leads to various bone diseases, including periodontitis [2–4]. Therefore, the reduction of osteoclast differentiation and/or resorbing activity could be an effective way to manage such bone diseases.

Osteoclastogenesis and the bone-resorbing activity of osteoclasts are mainly governed by the macrophage colony-stimulating factor (M-CSF) and receptor activator of nuclear factor-κB (RANK) ligand (RANKL) synthesized by osteoblasts/stromal cells [5, 6]. The interaction of RANK and RANKL triggers various signaling cascades that activate intracellular signaling molecules, including mitogen-activated protein kinases (MAPKs) and nuclear factor kappa-light-chain-enhancer of activated B cells (NF-κB). Subsequently, the signaling pathways lead to an increase in the expression of osteoclast-specific genes and bone resorption [7, 8].

Periodontitis is an inflammatory disease characterized by elevated alveolar bone resorption. Bacterial pathogens trigger a local inflammatory response, which stimulates the innate immune system [9, 10]. The initial local inflammatory response progresses to the gingival tissue through the action of an array of cytokines and mediators [9, 10]. The inflammatory response then drives the destruction of the alveolar bone by activated osteoclasts [11]. Before periodontitis progresses to the advanced stage where the only valuable treatment is tooth extraction, it is necessary to control alveolar bone loss. Howerer, therapeutic intervention for alveolar bone resorption is currently unavailable.

Pyrimidine, a well-known heterocyclic ring containing two nitrogen atoms, is found in both natural and synthetic compounds, such as nucleotides, thiamine, riboflavin, folic acid, and barbiturates [12]. It is known that pyrimidine derivatives and analogs exhibit extensive biological and pharmacological activity, including anti-bacterial, anti-HIV, anti-fungal, anti-inflammatory, anti-oxidant, and anti-cancer effects [13–15]. Because of these useful functions, a variety of pyrimidine derivatives have been synthesized and investigated as therapeutic agents. Vandyke and colleagues found that a tyrosine kinase inhibitor, dasatinib, which contains the pyrimidine ring, inhibited the bone-resorbing activity of osteoclasts, thus impairing bone metabolism [16]. In addition, an inhibitor of tyrosine-protein kinase BTK, ibrutinib, also containing the pyrimidine ring, ameliorated osteoclast-mediated bone destruction [17].

In this study, we synthesized novel pyrimidine derivatives, including OCLI-023, evaluated their capability to suppress RANKL-induced osteoclastogenesis and bone resorption, and investigated the molecular mechanisms. We also examined the effect of OCLI-023 on ligature-induced experimental periodontitis in mice.

## Materials and Methods

### Chemicals and reagents

**Fig 1:**
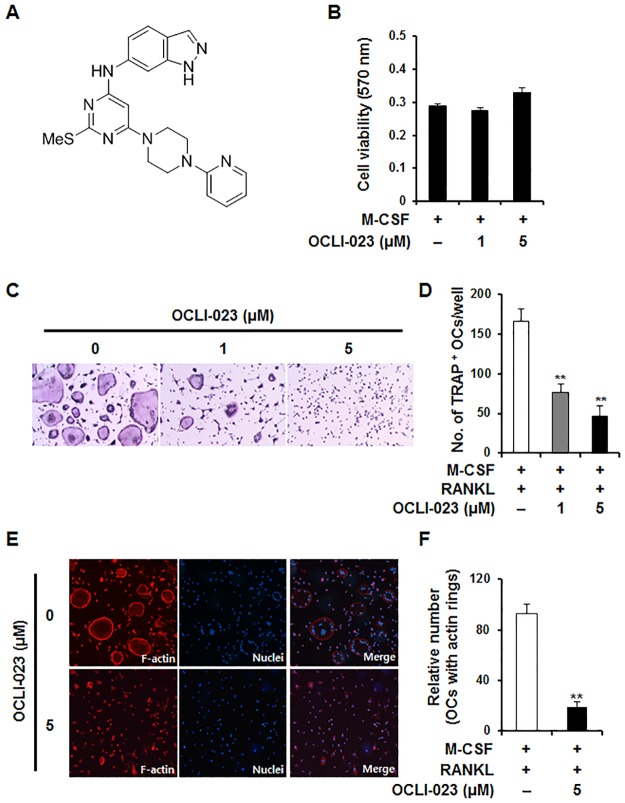
Effects of OCLI-023 on osteoclast differentiation in RANKL-stimulated BMMs. (A) Chemical structure of OCLI-023. (B) BMMs were incubated for 3 days with M-CSF (10 ng/mL) in the presence or absence of 1 μM or 5 μM OCLI-023. Cell viability was evaluated by the MTT assay. (C) BMMs were cultured with M-CSF (10 ng/mL) and RANKL (20 ng/mL) either with or without 1 μM or 5 μM OCLI-023. After 4 or 5 days, the cells were fixed and stained with TRAP. Magnification; 50X. (D) TRAP-positive multinucleated cells with ≥3 nuclei were scored. **p < 0.01 versus the vehicle-treated control. (E) BMMs were cultured on glass coverslips for 4 days with M-CSF (10 ng/mL) and RANKL (20 ng/mL) in the presence or absence of OCLI-023 (5 μM). The cells were stained with rhodamine-conjugated phalloidin and DAPI to visualize actin rings and nuclei, respectively. (F) The number of actin rings was analyzed. **p < 0.01 versus the vehicle-treated control.

Recombinant mouse M-CSF and mouse RANKL were purchased from R&D Systems (Minneapolis, MN, USA). OCLI-023, *N*-(2-(methylthio)-6-(4-(pyridin-2-yl)piperazin-1-yl)pyrimidin-4-yl)-1*H*-indazol-6-amine (Fig 1A), is a chemical compound that was synthesized by the addition of 1*H*-indazol-6-amine and 1-(pyridin-2-yl)piperazine to 4,6-dichloro-2-(methylthio)pyrimidine.

### Osteoclast differentiation

Osteoclast differentiation was performed as previously described [18]. Bone marrow cells obtained from 6- to 8-week-old C57B6/L mice (Dae Han Bio Link, Chungbuk, Korea) were incubated in α-minimal essential medium (α-MEM) containing 10% fetal bovine serum (FBS). After 24 h, non-adherent cells were centrifuged on a Histopaque density gradient (Sigma-Aldrich, St. Louis, MO, USA) and cultured in α-MEM supplemented with 10% FBS and M-CSF (30 ng/mL) for 3 days to obtain bone marrow macrophages (BMMs). BMMs were cultured with RANKL (20 ng/mL) and M-CSF (10 ng/mL) in the absence or presence of 1 μM or 5 μM OCLI-023 for 4 days. Then, the cells were stained with a tartrate-resistant acid phosphatase (TRAP)-staining solution prepared following the manufacturer’s instructions (Sigma-Aldrich). TRAP-positive multinucleated cells (MNCs), having three or more nuclei, were counted as osteoclast-like cells.

### Cell viability assay

The effect of OCLI-023 on the viability of BMMs was determined using the 3-(4,5-dimethylthiazol-2-yl)-2,5-diphenyltetrazolium bromide (MTT) assay (Sigma-Aldrich). BMMs were cultured with 1 μM or 5 μM OCLI-023 in the presence of M-CSF (10 ng/mL). After 3 days, MTT was added to each well, and the plate was incubated for 2 h. The formazan crystals were extracted with dimethyl sulfoxide, and the absorbance was measured at 570 nm using a 96-well microplate reader (BioRad, Hercules, CA, USA).

### Analysis of gene expression

Total RNA was isolated from cells using the TRI-solution (Bioscience, Seoul, Korea), and 1 μg of total RNA was reverse-transcribed using SuperScript II reverse transcriptase (Invitrogen, Carlsbad, CA, USA). Quantitative real-time polymerase chain reaction (PCR) was performed in a LightCycler 1.5 real-time PCR system (Roche Diagnostics, Rotkreuz, Switzerland) using TOPreal qPCR 2× PreMIX with SYBR green (Enzynomics, Daejeon, Korea). The amplification conditions consisted of an initial denaturation step at 95°C for 10 min, followed by 45 cycles of denaturation for 10 s at 95°C, annealing for 15 s at 60°C, and extension for 10 s at 72°C. The primers used for the PCR were as described previously [19].

### Immunoblot analysis

Whole-cell lysates were prepared using lysis buffer [50 mM Tris, pH 7.4, 150 mM NaCl, 1% NP-40, 1 mM ethylenediaminetetraacetic acid (EDTA), and protease and phosphatase inhibitors]. The protein concentration was measured with a BCA protein assay kit (Pierce Biotechnology, Rockford, IL, USA), and equal amounts of total protein (25 μg) were separated by 10% sodium dodecyl sulfate polyacrylamide gel electrophoresis. The separated proteins were transferred to nitrocellulose membranes (Whatman, Florham Park, NJ, USA). The membranes were incubated with 3% non-fat dry milk in TBS-T (25 mM Tris–HCl, pH 7.4, 150 mM NaCl, and 0.2% Tween 20) to block nonspecific binding. After blocking, the membranes were incubated with primary antibodies, followed by incubation with secondary antibodies. Proteins were detected using the WesternBright enhanced chemiluminescent substrate (Advansta, Menlo Park, CA, USA). The antibodies against phospho-JNK (#9251), phospho-IκBα (#2859), and IκBα (#9242) were obtained from Cell Signaling Technology (Danvers, MA, USA), and monoclonal anti-β-actin antibody (A5441) was purchased from Sigma-Aldrich (St. Louis, MO).

### Actin ring staining

Mouse BMMs were plated on glass coverslips in the presence or absence of 5 μM OCLI-023. After 4 days in culture, the cells were fixed with 4% paraformaldehyde, treated with 0.1% Triton X-100, and stained with rhodamine-conjugated phalloidin (Cytoskeleton, Denver, CO, USA) and 4′,6-diamidino-2-phenylindole dihydrochloride (DAPI; Santa Cruz Biotechnology, Santa Cruz, CA, USA) to visualize F-actin and nuclei, respectively. Fluorescent images were obtained using a BX51 fluorescence microscope (Olympus, Tokyo, Japan).

### Bone resorption assay

Mouse BMMs seeded on bone slices (IDS Nordic, Herlev, Denmark) were incubated with M-CSF and RANKL to induce osteoclast differentiation. After formation of multinucleated osteoclasts, the cells were incubated with or without 5 μM OCLI-023 for 2 more days. After incubation, all adherent cells were removed from the bone slices, and resorption pits were stained with Mayer’s hematoxylin solution. The area of resorbed pits was measured using the i-Solution image analysis software (IMT i-Solution, Daejeon, Korea).

### Ligature-induced alveolar bone loss model and histomorphometric analysis

All animal experiments were approved by the committees on the care and use of animals in research at Kyungpook National University and were conducted in accordance with the guidelines for the care and use of laboratory animals. To examine the efficacy of OCLI-023 *in vivo*, seven-week old male C57B6/L mice were assigned to the following four groups, with five mice in each: nonligation with vehicle (NL + V), nonligation with OCLI-023 (NL + OCLI-023), ligation with vehicle (L + V), and ligation with OCLI-023 (L + OCLI-023) groups. According to a previously published method, alveolar bone resorption was induced by ligation of 5–0 silk around the maxillary left second molar [20]. The vehicle or OCLI-023 (30 mg/kg) was injected to mice intraperitoneally for 7 days, starting on day 1. All mice were euthanized by carbon dioxide on day 8, and the maxillae were fixed in 4% paraformaldehyde. The fixed maxillae were scanned using a SkyScan 1272 high-resolution micro-computed tomography (μCT) system (Bruker, Kontich, Belgium) with a source voltage of 70 kV, current of 142 μA, and resolution of 6 μm. Three-dimensional images were obtained using the CTvox software, and to assess the alveolar bone loss, a linear distance from the CEJ to the ABC of the maxillary second molar was measured. For histological analysis, the fixed maxillae were decalcified with EDTA and embedded in paraffin. Sections (6 μm thick) were stained with hematoxylin and eosin (H&E) and TRAP.

### Statistical analyses

All experiments were conducted three times, and the data are presented as the mean ± standard deviation (SD). Statistical analyses were performed by the two-tailed Student’s *t*-test or one-way analysis of variance with Tukey’s multiple comparison post-hoc test. A *p* value of < 0.05 was considered statistically significant.

## Results

### Effects of OCLI-023 on viability of BMMs and on osteoclastogenesis

To examine the effect of OCLI-023 on the viability of osteoclast precursors (mouse BMMs), BMMs were cultured with M-CSF either with or without OCLI-023 (1 μM or 5 μM) for 3 days. We observed that neither low nor high concentrations of OCLI-023 affected the viability of BMMs compared with the control (Fig 1B). To elucidate whether OCLI-023 inhibits the RANKL-mediated osteoclast differentiation of BMMs, mouse BMMs stimulated with M-CSF (10 ng/mL) and RANKL (20 ng/mL) were incubated with or without 1 μM or 5 μM OCLI-023. As shown in Fig 1C, M-CSF and RANKL induced differentiation of BMMs into TRAP-positive MNCs (positive control), whereas treatment with OCLI-023 inhibited RANKL-stimulated osteoclast formation in a dose-dependent manner (Fig 1C). The number of TRAP-positive MNCs was reduced in a dose-dependent manner, and at 5 μM OCLI-023, the formation of MNCs was suppressed by 72.1% (*p* < 0.01) (Fig 1D).

We next examined the effect of OCLI-023 on the formation of both multinucleated cells and actin rings, which are morphological features of osteoclasts. Stimulation with M-CSF and RANKL led to cellular fusion into MNCs and rearrangement of the actin cytoskeleton (Fig 1E). However, OCLI-023 significantly inhibited the cellular fusion of osteoclast precursors and the formation of actin rings (Fig 1E and 1F).

### Effect of OCLI-023 on the expression of osteoclastic marker genes

**Fig 2:**
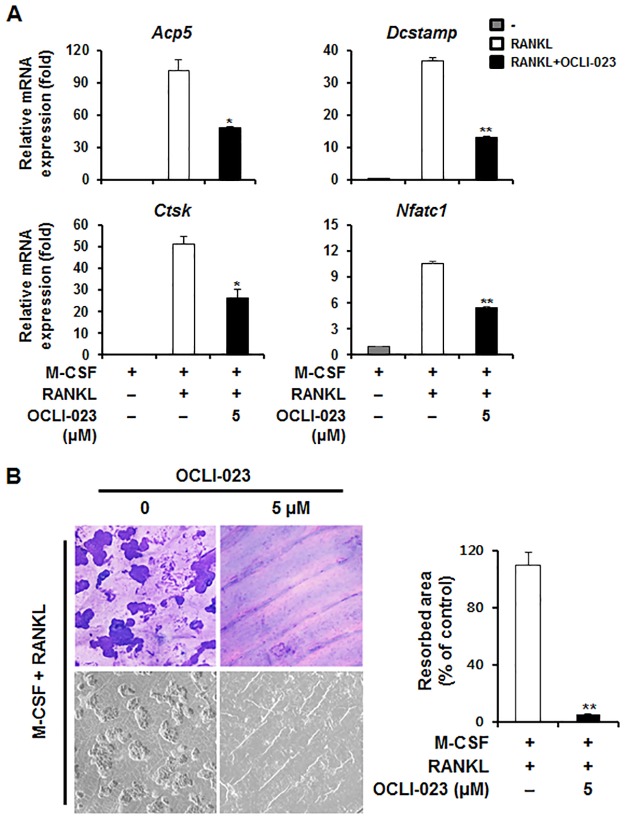
Effects of OCLI-023 on RANKL-induced mRNA expression during osteoclast differentiation and resorption pit formation. (A) BMMs were cultured in the presence of M-CSF (10 ng/mL) and RANKL (20 ng/mL) with vehicle or OCLI-023 (5 μM). The mRNA expression of TRAP (*Acp5*), cathepsin K (*Ctsk*), DC-STAMP (*Dcstamp*), and NFATc1 (*Nfatc1*) was analyzed using real-time quantitative PCR. (B) BMMs were seeded on bone slices and incubated with M-CSF (10 ng/mL) and RANKL (20 ng/mL) to induce osteoclast differentiation. After 3 days, the cells were incubated with or without OCLI-023 (5 μM) for an additional 2 days. Resorption pits were observed by hematoxylin staining (upper) and scanning electron microscopy (lower). **p < 0.01 versus the vehicle-treated control.

We investigated the mRNA expression of osteoclastic marker genes to confirm the effect of OCLI-023 on osteoclast differentiation. Expression of RANKL-induced nuclear factor of activated T-cells, cytoplasmic 1 (*Nfatc1*), encoding the central transcription factor for osteoclastogenesis (NFATc1), was attenuated by OCLI-023 (Fig 2A). Consistent with the decreased expression of *Nfatc1*, OCLI-023 suppressed the expression of osteoclast-specific genes, including *Acp5* (TRAP) and *Ctsk* (cathepsin K). In accordance with reduced cell–cell fusion and actin ring formation, expression of the *Dcstamp* gene encoding the dendritic cell-specific transmembrane protein (DC-STAMP), a chief regulator of cellular fusion of preosteoclasts, was downregulated by OCLI-023 (Fig 2A).

### Effect of OCLI-023 on bone resorption activity

As the major role of mature osteoclasts is to resorb mineralized tissue, we questioned whether OCLI-023 affects the resorbing activity of osteoclasts. To test this hypothesis, we generated osteoclast-like MNCs from BMMs on bone slices using M-CSF and RANKL. Then, the cells were incubated with or without OCLI-023 for an additional 2 days in an osteoclastogenic medium. The slices were stained with a hematoxylin solution, and we observed enlarged resorption pits in the positive control (Fig 2B). Treatment with OCLI-023 prevented the formation of resorption pits compared to the control (95% reduction), indicating that OCLI-023 significantly inhibits the resorbing function of osteoclasts (Fig 2B).

### Effect of OCLI-023 on RANKL-induced signaling pathways

**Fig 3:**
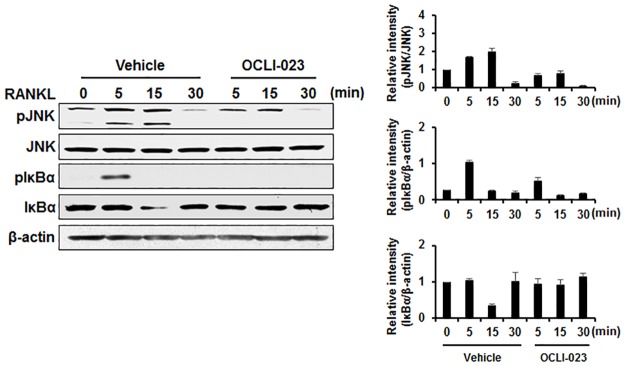
Effects of OCLI-023 on RANKL-stimulated signaling. BMMs were serum-starved for 5 h, then pretreated with OCLI-023 (5 μM) or vehicle for 1 h before RANKL (50 ng/mL) stimulation for the indicated times. Phosphorylation of JNK and IκBα was assessed by western blot. JNK or β-actin was used as the loading control.

The interaction between RANKL and RANK stimulates various signaling molecules essential for osteoclastogenesis and resorbing activity of mature osteoclasts. In particular, the MAPK and NF-κB signaling pathways have pivotal roles in osteoclast differentiation and function. To understand the underlying inhibitory mechanism of OCLI-023, BMMs were pretreated with OCLI-023 or the vehicle and stimulated with RANKL. Increased phosphorylation of JNK was observed in the vehicle-treated control, while RANKL-induced phosphorylation of JNK was reduced by treatment with OCLI-023 (Fig 3). In addition, phosphorylation and degradation of IκBα, induced by the RANKL stimulation, was abrogated when the BMMs were pretreated with OCLI-023, indicating that OCLI-023 attenuates the RANKL-induced osteoclastogenesis and resorbing activity by inhibiting the activation of the NF-κB signaling pathway, as well as phosphorylation of JNK (Fig 3).

### Effect of OCLI-023 on ligature-induced alveolar bone resorption

**Fig 4:**
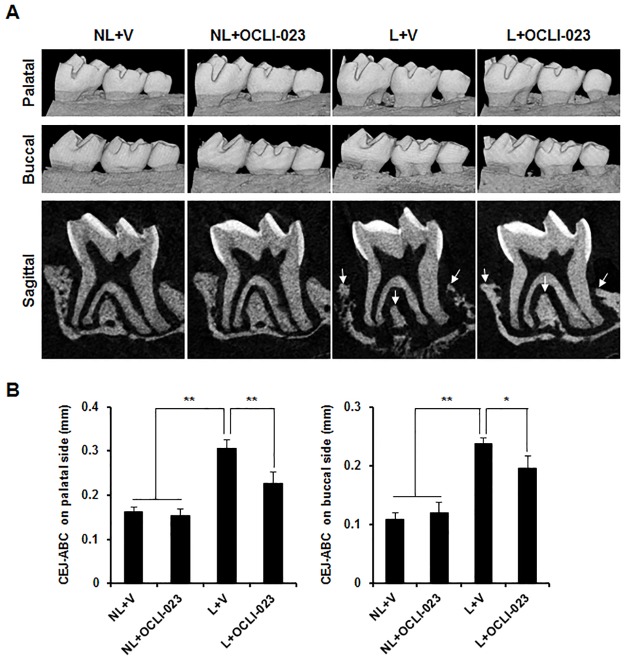
Micro-CT analysis of alveolar bones in mice with experimental periodontitis. (A) Both nonligated and ligated mice were injected with either vehicle or OCLI-023. Palatal (upper) and buccal (middle) sides of maxilla and sagittal (lower) sectional microCT images of the second molar exhibit the alveolar bone loss. NL + V, nonligated with the vehicle; NL + OCLI-023, nonligated with OCLI-023; L + V, ligated with the vehicle; L + OCLI-023, ligated with OCLI-023. (B) The linear distance from the CEJ to the ABC of the second molar was analyzed. n = 5 in each group. *p < 0.05, **p < 0.01 versus the ligated, vehicle-treated group.

**Fig 5:**
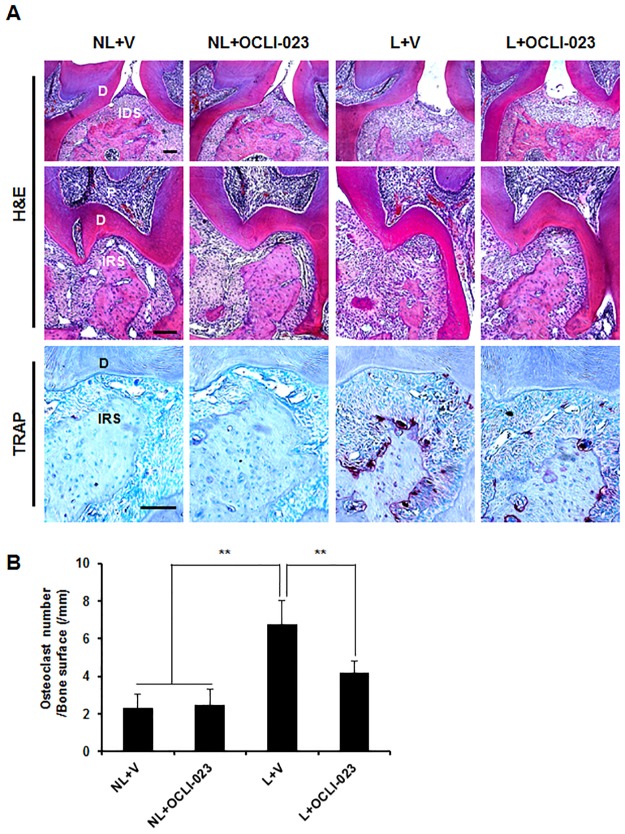
Histological analysis of alveolar bones in an experimental periodontitis model. (A) Both nonligated and ligated mice were injected with either vehicle or OCLI-023 as described in Fig 4. The fixed maxillae were decalcified, sectioned, and stained with H&E (upper and middle) and with TRAP (lower). Scale bars, 100 μm. P, pulp; D, dentin; IDS, interdental septum of the alveolar process; IRS, interradicular septum of the alveolar process. (B) The osteoclast number per bone surface was assessed. n = 5 in each group. **p < 0.01 versus the ligated, vehicle-treated group.

To examine the *in vivo* effect of OCLI-023 on periodontitis, the ligature-induced alveolar bone loss was assessed in mice. Both nonligated and ligated mice were administered with either vehicle or OCLI-023. The μCT images shown in Fig 4A revealed a serious alveolar bone loss of the maxillary left second molar in the ligated groups. However, the administration of OCLI-023 significantly reduced the bone resorption induced by the ligation (Fig 4A). The distance from the CEJ to the ABC of the molar on the palatal and buccal sides was decreased by 25.9% and 17.5%, respectively (Fig 4B). In addition, the interdental and interradicular septa of the alveolar process became eroded due to the ligature, but OCLI-023 reduced the ligature-induced alveolar process loss (Fig 4A, lower, arrows). Consistent with the μCT images, the histological sections stained with H&E showed the protective activity of OCLI-023 against alveolar bone resorption (Fig 5A). Resorption of both interdental and interradicular septa of the alveolar process, caused by the ligature, was substantially reversed by OCLI-023 (Fig 5A, H&E). The administration of OCLI-023 also suppressed the increase of the osteoclast number induced by the ligature, which suggested that OCLI-023 inhibits osteoclastogenesis and bone resorption *in vivo* (Fig 5A, TRAP and Fig 5B).

## Discussion

Anti-resorptive therapeutic agents including bisphosphonates can help prevent or slow the progression of skeletal diseases [21]. In spite of their beneficial effects, these medications can trigger various side effects, such as gastrointestinal problems and hypocalcemia [21, 22]. Hence, there have been extensive efforts to develop alternative medicines that can inhibit both osteoclast differentiation and the resorbing function of osteoclasts, without triggering any undesirable adverse events. In this study, we found that OCLI-023, a newly synthesized pyrimidine derivative, significantly inhibited the RANKL-mediated osteoclast differentiation in BMMs, as well as bone resorption of mature osteoclasts, by affecting the NF-κB and JNK signaling pathways. In addition, OCLI-023 suppressed alveolar bone loss induced by ligature in an *in vivo* model.

It is well known that the induction of NFATc1 expression is increased through the NF-κB, c-Fos, and calcium-calcineurin pathways activated by RANKL stimulation, and that NFATc1 is a principal regulator of RANKL-stimulated osteoclastogenesis [23]. Young mice with conditionally deleted *Nfatc1* exhibit osteopetrosis due to impaired osteoclast differentiation [24]. In addition, ectopic expression of NFATc1 leads to the differentiation of BMMs into osteoclasts without RANKL stimulation [25]. In our study, OCLI-023 attenuated the mRNA expression of *Nfatc1*, suggesting that OCLI-023 targets NFATc1 to interrupt RANKL-induced osteoclastogenesis (Fig 2A).

NFATc1 is capable of enhancing transcription of osteoclast-specific genes, such as *Acp5*, *Ctsk*, and *Dcstamp* [25]. Downregulation of *Nfatc1* expression by OCLI-023 treatment led to decreased expression levels of the osteoclast-specific genes (Fig 2A). Among these genes, *Dcstamp* encodes a pivotal factor for cellular fusion during osteoclast differentiation [26]. We found that the cell–cell fusion of osteoclast precursors and actin ring formation were impaired by OCLI-023, suggesting that the important osteoclastogenic processes of multinucleated giant cell formation and cytoskeletal rearragements were suppressed by OCLI-023 through the inhibition of *Dcstamp* expression.

The RANKL-stimulated MAPK signaling pathways regulate the transcription of genes involved in osteoclast differentiation [2]. In particular, Ikeda and colleagues reported that the c-Jun signaling cascade performed an essential function in both RANKL-induced osteoclastogenesis and the regulation of osteoclast apoptosis [27, 28]. OCLI-023 considerably attenuated the JNK phosphorylation induced in response to RANKL (Fig 3). The NF-κB signaling pathway is also activated by RANKL stimulation, and several studies of genetically modified mice have revealed that the NF-κB pathway is crucially involved in osteoclast differentiation. The deletion of NF-κB p50 and p52 results in osteopetrosis in mice owing to the defects in osteoclast formation [29]. In addition, the IκB kinase beta, which is required for phosphorylation of IκBα and subsequent NF-κB activation, is essential for osteoclast differentiation [30]. We observed that OCLI-023 blocked the RANKL-induced phosphorylation and degradation of IκBα (Fig 3). Taken together, these results indicate that OCLI-023 exerts its inhibitory effects on osteoclast differentiation and the bone-resorbing function by suppression of the JNK and NF-κB signaling pathways.

As pyrimidine derivatives exhibit a wide range of biological and therapeutic activities, pyrimidine and its derivatives have been the subjects of many investigations [31–33]. A thienopyrimidine compound, NSL-1406, inhibits the bone-resorbing function of osteoclasts as a result of its cytotoxic activity on osteoclasts [34]. Substituted 5,7-diphenyl-pyrrolo[2,3-*d*]-pyrimidines lead to osteoclast apoptosis through the sustained activation of extracellular signal-regulated kinases [35] and c-Src kinase [36]. Unlike these derivatives, OCLI-023 exhibits inhibitory action on both osteoclastogenesis and the resorbing activity of mature osteoclasts, with no substantial cytotoxicity (Fig 1). We believe that different effects of OCLI-023 on osteoclasts from other pyrimidine derivatives arise because it may target different signaling molecules/pathways such as JNK and NF-kB in RANKL signaling pathways (Fig 3).

Periodontitis is an osteolytic bone disease that leads to the destruction of alveolar bones around the teeth and, ultimately, the loss of teeth. Because periodontitis progress is closely associated with enhanced osteoclast formation and/or bone-resorbing activity, the inhibition of osteoclast formation and/or osteoclastic bone resorption may be an effective strategy for the therapeutic intervention for periodontitis. In addition to the inhibitory effect of OCLI-023 on osteoclast differentiation and function *in vitro*, the treatment with OCLI-023 also considerably attenuated the alveolar bone destruction in a mouse model of experimental periodontitis (Figs 4 and 5). OCLI-023 protected both interdental and interradicular septa of the alveolar processes from ligature-induced alveolar bone loss, suggesting that the osteoclast formation and activation are substantially inhibited by OCLI-023. TRAP staining demonstrated that the osteoclast numbers were significantly reduced by OCLI-023 (Fig 5A, lower and Fig 5B). These results suggest that OCLI-023 can suppress alveolar bone resorption by targeting osteoclasts, however, we could not exclude the possibility that OCLI-023 may also regulate osteoblast differentiation and formation [37]. Furthermore, the limitation of this study is that direct target molecule of OCLI-023 is undefined.

In conclusion, the present study showed that OCLI-023, a newly synthesized pyrimidine derivative, inhibited RANKL-induced osteoclastogenesis and the resorbing activity of osteoclasts via suppression of the JNK and NF-κB pathways *in vitro* and reduced the alveolar bone loss *in vivo*. Therefore, we suggest that OCLI-023 may have the potential to improve bone quality and/or prevent bone loss.
